# Ensemble-based classification approach for PM2.5 concentration forecasting using meteorological data

**DOI:** 10.3389/fdata.2023.1175259

**Published:** 2023-06-09

**Authors:** S. Saminathan, C. Malathy

**Affiliations:** ^1^Department of Computing Technologies, Faculty of Engineering and Technology, SRM Institute of Science and Technology, Kattankulathur, Tamil Nadu, India; ^2^Department of Networking and Communications, Faculty of Engineering and Technology, SRM Institute of Science and Technology, Kattankulathur, Tamil Nadu, India

**Keywords:** air quality forecast, supervised machine learning, multiclass classification, imbalanced data set, SMOTE

## Abstract

Air pollution is a serious challenge to humankind as it poses many health threats. It can be measured using the air quality index (AQI). Air pollution is the result of contamination of both outdoor and indoor environments. The AQI is being monitored by various institutions globally. The measured air quality data are kept mostly for public use. Using the previously calculated AQI values, the future values of AQI can be predicted, or the class/category value of the numeric value can be obtained. This forecast can be performed with more accuracy using supervised machine learning methods. In this study, multiple machine-learning approaches were used to classify PM2.5 values. The values for the pollutant PM2.5 were classified into different groups using machine learning algorithms such as logistic regression, support vector machines, random forest, extreme gradient boosting, and their grid search equivalents, along with the deep learning method multilayer perceptron. After performing multiclass classification using these algorithms, the parameters accuracy and per-class accuracy were used to compare the methods. As the dataset used was imbalanced, a SMOTE-based approach for balancing the dataset was used. Compared to all other classifiers that use the original dataset, the accuracy of the random forest multiclass classifier with SMOTE-based dataset balancing was found to provide better accuracy.

## Introduction

Air pollution is a widespread problem worldwide. Air pollution is the result of contamination of both ambient and indoor environments (World Health Organization, 2022a) (WHO). Major outdoor pollutants are particulate matter under 10 μm (PM10), particulate matter under 2.5 μm (PM2.5), ammonia (NH3), carbon monoxide (CO), nitrogen dioxide (NO2), and ozone (O3).

The necessity to computate air pollution rises because of the serious health effects it has on human beings. The major sources and harmful effects of air pollution have been summarized in previous studies (Leon et al., 1996; Han et al., 2010; Swarna Priya and Sathya, 2019; Singh et al., 2020; Puja, 2022). The harmful effects include respiratory illnesses and other diseases affecting humans as well as global warming. Sulfur dioxide is produced during fuel combustion in power plants and by vehicle emissions and during solid refuse disposal. It causes respiratory issues, headaches, and decreased agricultural output. Road, rail, and commercial transportation, as well as the use of industrial boilers and fuel combustion, all produce nitrogen dioxide. It damages the leaves and causes respiratory issues. Transportation and the burning of waste produce carbon monoxide. As a result, breathing activities are affected, and headaches are also common. Ozone (O3) is a pollutant that results from photochemical processes. It causes premature aging, necrosis, and bleaching. Burning fossil fuels and clearing forests produce carbon dioxide, and the result is global warming. The sources of PM10 and PM2.5 are domestic wood, building activities, road dust, and diesel vehicle exhaust, which have toxic effects on humans.

Sustainable Development Goals (SDGs) 3, 7, and 11 envisaged by the (United Nations General Assembly Resolution, 2015) WHO are related to air pollution. The WHO tracks and monitors health indicators, utilizing its technical knowledge to determine how far these goals have been achieved (World Health Organization, 2022b) (WHO).

To control air pollution, the air quality index must be calculated first. This can be calculated as the worst sub-index value of all the pollutants. The value can be analyzed for making corrective measures. In this study, various machine learning methods were used to classify the PM2.5 levels on an air quality dataset. Along with logistic regression, SVM and one from each of the bagging and boosting types (random forest and XGBoost) with their grid search equivalents and an artificial neural network (multilayer perceptron) have been used for PM2.5 multiclass classification. A random forest multiclass classifier with SMOTE-based dataset balancing is also proposed. Accuracy and per-class accuracy were used to compare these methods.

### Air quality index

The air quality index (AQI) is a measure that can provide qualitative information on air quality and potential health consequences. The definition of the air quality index (AQI) differs according to different standards-making institutions globally. In India, the National AQI program was developed by India's Central Pollution Control Board and presented in a report (Central Pollution Control Board, 2015) (CPCB).

If data for at least three of the pollutants are available, the AQI can be calculated. PM 2.5 or PM 10 should be included in the data for these three pollutants. A subindex was created for each pollutant based on the measured values, relevant standards, and possible health effects induced by the pollutant. The total AQI was calculated using the worst of these subindices.

In India, the AQI computation is based generally on eight pollutants (Central Pollution Control Board, 2015) (CPCB). They are PM10, CO, PM2.5, NO2, O3, SO2, NH3, and Pb. There are six possible AQI classes, ranging from good to severe. Table 1 shows eight pollutant parameters and their corresponding AQI breakpoints (Central Pollution Control Board, 2015).

**Table 1:** Pollutant parameters and their corresponding AQI breakpoints.

**AQI Category (Range)**	**PM10**	**PM2.5**	**NO2**	**O3**	**CO**	**SO2**	**NH3**	**Pb**
	**24-h**	**24-h**	**24-h**	**8-h**	**8-h (mg/m** ^3^ **)**	**24-h**	**24-h**	**24-h**
Good (0–50)	0–50	0–30	0–40	0–50	0–1.0	0–40	0–200	0–0.5
Satisfactory (51–100)	51–100	31–60	41–80	51–100	1.1–2.0	41–80	201–400	0.6 - 1.0
Moderate (101–200)	101–250	61–90	81–180	101–168	2.1–10	81–380	401–800	1.1–2.0
Poor (201–300)	251–350	91–120	181–280	169–208	10.1–17	381–800	801–1,200	2.1–3.0
Very poor (301–400)	351–430	121–250	281–400	209–748^*^	17.1–34	801–1,600	1201–1,800	3.1–3.5
Severe (401–500)	430+	250+	400+	748+^*^	34+	1,600+	1,800+	3.5+

Breakpoints for the AQI scale 0–500 (units: μg/m^3^ except if stated otherwise).

* One hourly monitoring for O3.

For example, if the range of the PM2.5 value is 61–90, the AQI category for PM2.5 is considered moderate. Then, for subindex calculation, the AQI value would be converted to the range 101–200 from the range 61–90 for PM2.5 by normalization. If any PM2.5 value is above 250, the air quality category for PM2.5 is considered severe. For AQI calculations, the values of all the pollutants are converted to their numeric subindex values, and the worst index of all pollutants is used as the air quality index.

## Related works

The methods for predicting and classifying the air quality index were examined in this study. In addition, the approaches for dealing with the imbalance in datasets are outlined below.

In their research, Babu Saheer et al. (2022) focused on obtaining solid evidence that might be used to make better climate change policy proposals. One of the best ways to combat the issue of climate change is to watch, predict, and oversee the air quality in urban areas, which can be done by utilizing the large volume of data that is available in areas, such as pollutant concentration, city traffic, aerial photography of topography and vegetation, and climate conditions. Using cutting-edge artificial intelligence techniques, this research presents an innovative, economical, and efficient framework for modeling air quality that considers all these elements. The framework was used in the city of Cambridge. Models such as SVR with various kernels, statistical ARIMA models, various linear and non-linear regression approaches, and an advanced LSTM-based deep learning model were used. The SVR models produced encouraging results. The deep learning models exhibited some potential for advancement with better data and optimization.

In their study, Mendes et al. (2022) described a statistical method for forecasting air quality that accurately predicts PM10, PM2.5, NO2, and O3 values for the following day. The assessment measures used demonstrated the effectiveness of the stated statistical technique for air quality forecasting.

The daily 72-h O3 forecasts were produced using the ML modeling framework proposed by Fan et al. (2022). In this study, the machine learning models were used to create forecast systems that are more accurate while requiring significantly less processing using the past meteorological dataset (temperature, relative humidity, etc.) and O3 observation data. The new forecast framework is comprised of two ML models, namely, ML1 and ML2, which forecast the O3 mixing ratios and AQI classifications. ML1 uses both the random forest classifier and MLR models. ML2 uses a two-phase random forest regression model with weighting components. The ML modeling approach has been demonstrated to be more effective than chemical transport models in predicting ground-level O3.

Li et al. (2011) assessed the spatial flow variation of air pollutants in the Chengdu city region. The forecast maps of toxin fixations produced by the OK method were superior to the IDW technique.

Decision trees, random forest, multilayer perceptrons, and gradient boosting were used to predict pollution in the study by Ameer et al. (2019). The experiments were carried out using Apache Spark. According to the results obtained, the random forest method outperformed other methods. PM2.5 was the pollutant considered in the study. In addition, an architecture for predicting air pollution has been outlined. In addition, the study includes a correlation matrix of PM2.5 with four meteorological variables such as dew and wind speed. RMSE and MAE comparison graphs for the five cities considered were also included.

A comparative assessment of various machine learning methods for air quality prediction was carried out in the study by Ameer et al. (2019) and Senthivel and Chidambaranathan (2022).

A prediction method based on environmental data gathered from Shanghai and deep learning methods was proposed in the study by Qin et al. (2019). To automatically obtain the features of the data given as input, CNN was used. An LSTM network was used as the output layer. After convolution and pooling, the spatial correlation between the data was estimated. The authors used features such as PM2.5 concentration, temperature, humidity, wind speed, precipitation, wind direction, and other pollutant concentrations as input to the model. The proposed model performed the best compared with BP, CNN, RNN, and LSTM in terms of RMSE and correlation coefficient.

Deep learning was used to predict ozone concentrations in the study by Ghoneim and Doreswamy (2017). DNN showed increased accuracy when compared to GLM, NN, and SVM.

In the study of Sakarkar et al. (2020), the authors compared seven machine learning methods and found that the boosted random forest algorithm provides precise predictions for pollution data of Nagpur.

Liu et al. (2019) forecasted Beijing's AQI values and calculated the NOX levels in an Italian city using support vector regression (SVR) and random forest regression (RFR). The SVR-based model performed well in foretelling the AQI. The RFR-based model performed well in forecasting the NOX levels.

Chang et al. (2020) proposed a model called ALSTM (aggregated LSTM model). A composite model for prediction was created by combining three LSTM models. Data from Taiwan's Environmental Protection Agency was used in this study. To forecast PM2.5, the authors found that the proposed model helped increase prediction accuracy compared to the other models.

The naive Bayes algorithm and a decision tree (J48) were used in the study by Gore and Deshpande (2017) in the classification of air quality. The accuracy of the decision tree was ~5% higher than the other one. Comparative graphs based on the measures of sensitivity, FP rate, precision, and recall were also used.

Mahalingam et al. (2019) discussed the issues in predicting the air quality index (AQI), and AQI data for Delhi were considered. The findings demonstrated that the suggested model using ANN followed by SVM had higher prediction accuracy than the other models.

An effective method was used to construct the predictive air quality map for Tehran's next 24 h, as given in the study by Asgari et al. (2017). The authors used logistic regression and Apache Hadoop with naive Bayes. They found the most accurate estimator to be logistic regression. Class prediction using logistic regression is used to create the predictive air quality risk map.

Sharma et al. (2019) investigated the trends in air pollution in three Indian cities. It discussed the annual growth/decline of pollutants such as SO2, NOx, and PM2.5 from 2015 to 2018.

Sports facilities (SFs) require substantial amounts of energy. To address the issues of sustainability and efficiency in such facilities, intelligent and successful solutions are needed. A systematic literature study that examines the development of SF operations, sustainability, and energy optimization is provided in the study by Elnour et al. (2022a). Compared to residential and commercial buildings, few studies have been done on the management and optimization of SFs in the last 5 years, and 71% of them were for facilities in cold climates. The study addressed the research gap between sports facilities and other buildings in terms of management. It stressed the significance of regional context for sports facility operation management.

Detailed research on AI and big data analytics for building automation and management systems was performed by Himeur et al. (2022a), describing current issues and prospective solutions. They described how clustering can be used, among other things, to detect and monitor air pollutants. They have identified how the cooks and kitchen staff are exposed to air pollution created by cooking and the resulting health issues. The authors have conducted a thorough analysis of pollutants and indoor environmental quality.

Sports facilities, unlike other buildings, have high energy requirements. As a result, managing and optimizing them is essential for lowering their energy usage and carbon footprint while preserving a suitable indoor environmental quality. Elnour et al. (2022b) recommended a technique that comprises a prediction element and an optimizer and offers an integrated dynamic optimization method that takes into consideration potential system behavior in the decision-making process. The study of these authors also outlines how to maintain the air quality in terms of CO_2_ levels at safe and healthy levels inside the facilities.

The research by Himeur et al. (2022b) provided a thorough analysis of transfer learning (TL)-based energy systems, which has a big impact on creating next-generation energy systems for applications in smart cities. TL has been credited with successes, including load forecasting, thermal comfort control, smart grid, and energy trading. Through a discussion of current frameworks, their learning processes, and their advantages and disadvantages, the authors outline in this framework the substantial progress made by the TL community in energy systems. They have focused on the shortcomings of traditional machine learning algorithms and how transfer learning may help them perform better in energy systems. Overall, this analysis will serve as a thorough reference for the research community on energy and smart cities with TL-based energy systems.

The goal of the research by Aguilera et al. (2023) was to isolate wildfire-specific PM2.5 from other sources of emissions using statistical techniques. The study first presented an ensemble model that optimally merges multiple machine learning methods, such as gradient boosting machines, deep learning, and a large set of explanatory variables. Then, a statistical method was used to calculate the PM2.5 concentrations unique to wildfires, which may be used in any geographic area.

Employing PM2.5 as an example scenario, Ejohwomu et al.'s (2022) study examined the efficacy of employing ensemble models for predicting the amounts of air pollutants compared to that of other standalone algorithms. The generated models demonstrated the effectiveness of machine learning algorithms in forecasting air particle concentrations and their potential for use in air pollution control. This study showed the way the meteorological variables, temperature and relative humidity, affect the levels of contaminants in the air.

To forecast the value of PM2.5, the research by Yin et al. (2022) recommended an ensemble architecture that integrates four different learning models. The feature set includes variables such as periodic, meteorological, and autoregression variables. In this study, the experimental dataset from Puli Township in central Taiwan, which covers the interval from 1 January 2008 to 31 December 2019, was used. The experimental findings demonstrated that the recommended multimodel framework might effectively combine the benefits of embedded models to produce a better-predicting outcome.

By modeling the target city's air and climatic variables, as well as the PM2.5 values of its neighboring cities, the authors in the study by Yu et al. (2022) built a multi-scale ensemble learning method in this work to estimate the daily PM2.5 concentrations of the target city. First, the suggested method performs multifeature selection that is based on the distance factor and predictive capacity of data after smoothing the multivariate data using singular spectrum analysis. Second, multivariate empirical mode decomposition captures the natural relationship between the acquired multiple features. Third, for multistep prediction, the Hurst exponent is used to link each time scale with the associated predictor. The PM2.5 value forecasting results are then obtained by adding the predicted data from all time frames. To test the precision and reliability of the suggested method, four experiments were conducted in Beijing, Wuhan, and Shenzhen. The results demonstrated that this approach performs better than all benchmark ones.

In the study by Zheng et al. (2023) the authors presented a deep learning model that is driven by the wavelet-packet transform (WPT) to forecast the hourly PM2.5 concentration and tested its efficacy in Qingdao, China. The meteorological data were initially divided into sub-time series with various frequencies and resolutions using the wavelet packet. A multidimensional LSTM that considers both spatial and temporal information was created. This helps in PM2.5 prediction. The method proposed in this study has better PM2.5 prediction performance compared to other methods.

To estimate mean and maximum PM2.5 values in the Mexico City Metropolitan Area from 2004 to 2019, Gutiérrez-Avila et al. (2022) set out to create a machine-learning model. They describe a novel modeling strategy that uses aerosol optical depth, land use, and meteorology variables and is based on extreme gradient boosting (XGBoost) and inverse distance weighting. The models to calculate the mean and maximum of PM2.5 performed well. The concentrations of PM2.5 were higher on hotter days.

The public can be alerted about the rising PM2.5 concentration using an accurate and effective forecasting technique as described in the study by Li et al. (2023). The AdaBoost-ensemble technique was proposed in this research. A novel decomposition technique based on the hybrid data preprocessing-analysis approach was recommended. A novel AdaBoost-LSTM ensemble technique was then developed to integrate the individual forecasting results into the final forecasting results, which significantly improves forecasting performance. The forecasting results were then combined. The experimental findings have shown that the developed hybrid model significantly outperformed the compared models.

To offer a novel CNN-RF ensemble framework for PM2.5 concentration modeling, the study by Chen et al. (2023) combined the benefits of convolutional neural network (CNN) feature extraction and the regression capability of random forest (RF). For model training and testing, data from 13 monitoring sites in Kaohsiung were chosen in 2021. In the beginning, CNN was used to extract important meteorological and environmental data. Apart from air pollution factors, six meteorological factors, such as wind speed, rainfall, wind direction, relative humidity, and ambient temperature, and four spatiotemporal factors, such as day, hour of the day, and spatial coordinates, were all included in each observation. Then, using five input factors, to extract features from the CNN and spatiotemporal factors, the random forest algorithm was used to train the model. The results have highlighted that the suggested CNN-RF model could model the data more accurately than the individual CNN and RF models.

Concentrations of particulate matter (PM2.5) are primarily influenced by pollution emissions and weather conditions. Based on a high-resolution atmospheric composition reanalysis dataset given for 4-years, Kou et al. (2021) examined the meteorological influence on better PM2.5-related air quality between 2016 and 2019 in China. The correlation between weather and air quality has been further explored using the objective weather classification method. The findings showed that, in conjunction with China's stringent enforcement of its clean air policy from 2016 to 2019, meteorological conditions have contributed positively to improvements in air quality.

In their study, Alpan and Sekeroglu (2020) predicted six pollutant levels using machine learning algorithms with meteorological data such as precipitation and temperature. The random forest had a high prediction ability with experiments on two different datasets. The authors claimed that high-accuracy forecasts on pollutant concentrations could be performed using only meteorological data.

Meteorological factors influence air quality. The studies Ameer et al. (2019), Qin et al. (2019), Alpan and Sekeroglu (2020), Kou et al. (2021), Ejohwomu et al. (2022), Fan et al. (2022), Gutiérrez-Avila et al. (2022), Chen et al. (2023), and Zheng et al. (2023) have included meteorological data and air pollution data.

### Approaches for handling an imbalanced dataset

A general problem that can occur with respect to the dataset when multiclass classification is used is that the datasets can be imbalanced. In imbalanced datasets, a few classes tend to have more data items, and a few classes have very limited data. This may affect the accuracy of the results. The imbalance in class can be tackled using many approaches. The Synthetic Minority Over-sampling TEchnique (SMOTE)-based approach for improving the accuracy of the algorithms for imbalanced datasets has been discussed in the research by Chawla et al. (2002), Bahaweres et al. (2022), Pribadi et al. (2022), and Karagöl et al. (2022).

The SVM, RF, NB, and NN classification algorithms were used by Bahaweres et al. (2022) to classify data from five separate NASA datasets. The SMOTE has been shown to mitigate the impact of class imbalance. The g-mean scores across all models were found to have improved.

Using Twitter data regarding the Sinovac vaccine on an imbalance class, Pribadi et al. (2022) conducted a sentiment analysis and discovered that, after SMOTE optimization, the accuracy values of the three methods they employed, on average, increased by 14%.

Karagöl et al. (2022) classified darknet traffic using six different machine learning methods, both with and without SMOTE, and found that the SMOTE approach produced the highest accuracy results.

A summary of some of the machine learning and deep learning methods outlined above that were used for PM2.5/air pollutant forecasting is given in Table 2.

**Table 2:** Summary of the machine learning methods for PM2.5/air pollutant forecasting.

**Sl. No**.	**References**	**Location/datasets**	**Pollutants and other variables considered**	**Methods used**	**Inference**
1	Ameer et al. (2019)	Five cities in China	PM2.5, meteorological	DT, Random Forest, MLP, Gradient Boosting	RF was found to be the best method
2	Qin et al. (2019)	Shanghai	PM2.5	CNN, LSTM	The proposed model performs better
3	Sakarkar et al. (2020)	Nagpur	SO2, NO2, RSPM, SPM, PM2.5	7 algorithms	Boosted RF algorithm yields an accurate prediction
4	Chang et al. (2020)	Data provided by Taiwan Environmental Protection Agency	PM2.5	Aggregated LSTM model	The accuracy of prediction improved
5	Aguilera et al. (2023)	US	PM2.5	A statistical method	Can isolate wildfire-related PM2.5
6	Ejohwomu et al. (2022)	Lagos	PM2.5, meteorological	Ensemble method	Correlation between meteorological variables and contaminants
7	Yu et al. (2022)	Beijing, Wuhan, and Shenzhen	PM2.5 of the given city, climatic variables, and PM2.5 of cities nearby	Multi-scale ensemble learning	The approach performs better than all benchmark ones
8	Zheng et al. (2023)	Qingdao	PM2.5, meteorological	A deep learning model with the wavelet-packet transform	The method gives better PM2.5 prediction performance compared to other ones
9	Gutiérrez-Avila et al. (2022)	Mexico City Metropolitan Area	PM2.5, aerosol optical depth, land-use and meteorological	XGBoost and inverse-distance weighting	The models to calculate the mean and maximum of PM2.5 performed well. The values of PM2.5 were higher on hotter days.
10	Li et al. (2023)	Shaanxi province	PM2.5	AdaBoost-ensemble method	The hybrid model significantly outperformed the compared models
11	Chen et al. (2023)	Kaohsiung	Meteorological, air pollution, spatiotemporal	CNN, RF	The results have highlighted that the suggested CNN-RF model could model the data more accurately than the individual CNN and RF models.
12	Kou et al. (2021)	High-resolution atmospheric composition reanalysis dataset	PM2.5	Objective weather classification method	Meteorological conditions have contributed positively to improvements in air quality with the enforcement of a clean air policy
12	Alpan and Sekeroglu (2020)	Beijing multi-site air-quality data dataset	Meteorological data	Random forest	High-accuracy forecasts on pollutant concentrations can be performed using only meteorological data
13	Bahaweres et al. (2022)	Five NASA datasets	–	The SVM, RF, NB, and NN classification algorithms	The SMOTE mitigated the impact of class imbalance
14	Pribadi et al. (2022)	Twitter data regarding the Sinovac vaccine	–	Sentiment analysis	After SMOTE optimization, the accuracy values, on average, increased by 14%.
15	Karagöl et al. (2022)	Darknet traffic	–	Three classification approaches	The SMOTE approach produced the most accurate results
16	Elnour et al. (2022a)	–	–	A systematic literature study that examines the development of SF operations, sustainability, and energy optimization is provided	The work addressed the research gap between sports facilities and other buildings in terms of management.
17	Himeur et al. (2022a)	–	–	Detailed research on AI and big data analytics for building automation and management systems	Identified how the cooks and kitchen staff are exposed to air pollution due to cooking and the resulting health issues. A thorough analysis of pollutants and Indoor Environmental Quality was done
18	Elnour et al. (2022b)	–	–	A technique that consists of a prediction element and an optimizer	It outlines how to maintain the air quality in terms of the CO_2_ level to be maintained at safe and healthy levels inside the sports facilities.
19	Himeur et al. (2022b)	–	–	Provided a thorough analysis of transfer learning based energy systems	This analysis can serve as a thorough reference to help the research community on energy and smart cities with TL-based energy systems.

## Proposed system

In the study proposed here, the air pollution database for the 12 cities of China was considered, and an attempt was made to classify the PM2.5 levels using various machine learning methods. For multiclass classification, two basic algorithms, such as logistic regression and support vector machine, and one each from bagging (random forest) and boosting (extreme gradient boosting) types, were used. These four algorithms were used along with their grid search counterparts to make them eight in number. An artificial neural network (multi-layer perceptron) was also used for classification. The parameters, such as accuracy and per-class accuracy, were used to compare all the machine learning methods. In addition, the algorithm that would provide more accuracy by tuning various hyper-parameters has been found for the pollutant PM2.5. This can be extended to all the pollutants for which data are available.

**Figure 1:**
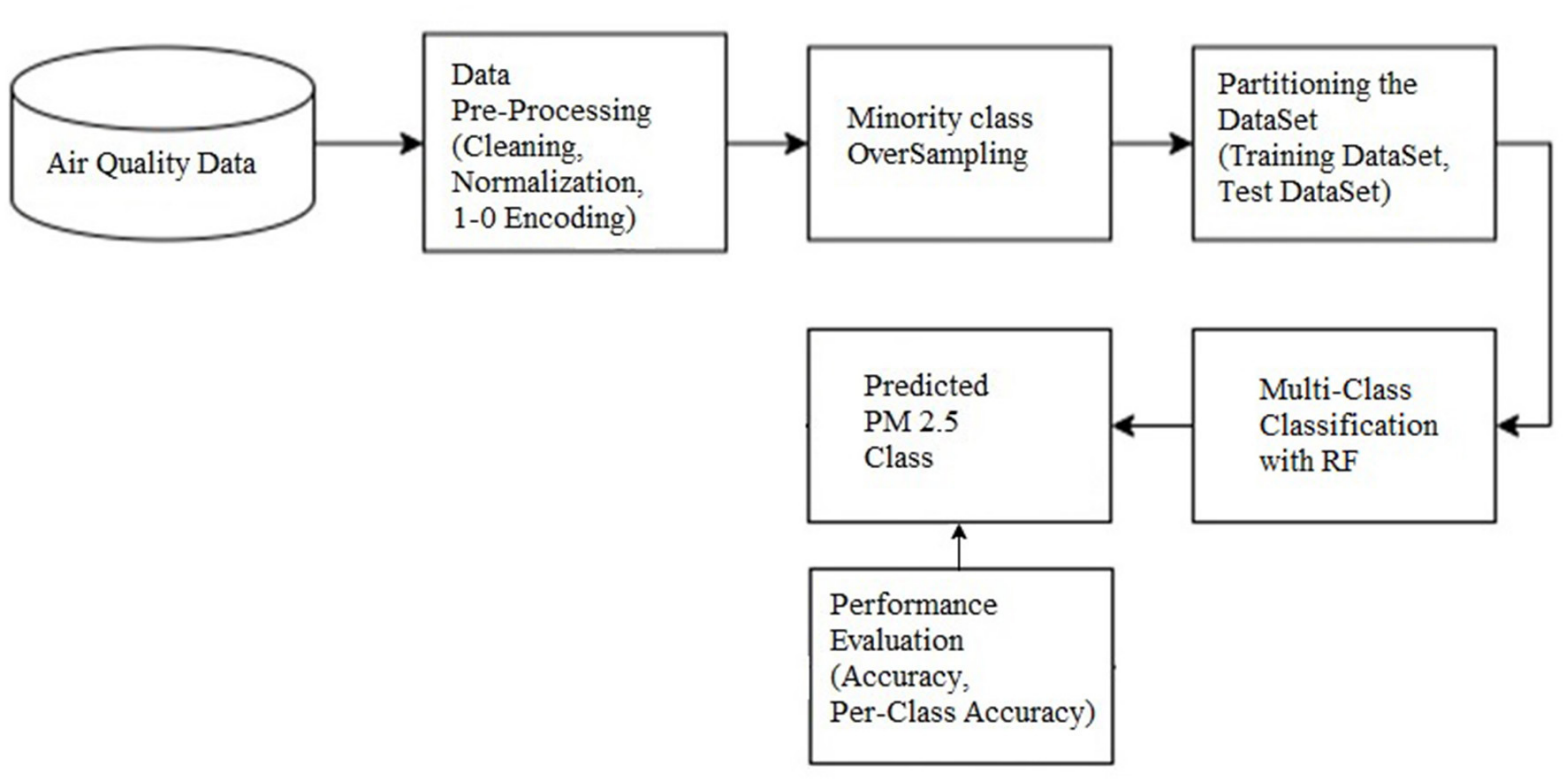
Workflow diagram of the proposed system.

The random forest-based ensemble method provides greater classification accuracy. In addition, as the dataset used was imbalanced, a SMOTE-based approach for balancing the dataset was used. The accuracy of the random forest multiclass classifier with SMOTE-based dataset balancing was found to provide better accuracy compared to all other classifiers that use the original dataset, and the results are discussed. Figure 1 outlines the workflow of the proposed system.

Most machine learning models have hyper-parameters that can be changed to alter the model's learning process. Grid search uses many values for the hyper-parameters and chooses the one that results in the best score and applies it to the model.

**Figure 2:**
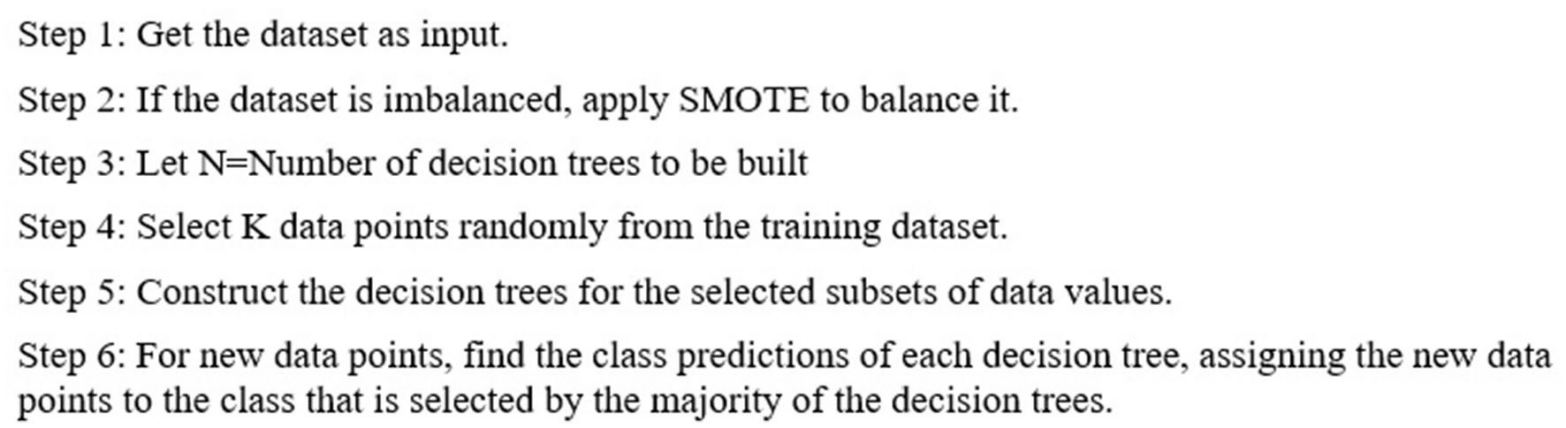
RF-SMOTE algorithm used.

The proposed RF-SMOTE algorithm is outlined in Figure 2.

The input dataset was obtained from the user. If it was imbalanced, it was corrected using the SMOTE approach. N decision trees were combined to form the random forest, and then, predictions were produced for each tree. New points were assigned to the class that received the majority of votes by the trees.

### Dataset description

The data provided in (UCI Machine Learning Repository, 2017)(UCI ML repository) was used in this study. This dataset comprises 17 attributes, including year, month, day, hour, PM2.5, PM10, SO2, NO2, CO, O3, temperature, pressure, wind direction, and dew point. It consists of hourly air pollutant data recorded for 12 air-quality monitoring stations from Aotizhongxin to Wanshouxigong in the PRC for the period 01 March 2013 to 28 February 2017.

The above dataset was considered for the following reasons. The number of records was close to 0.4 million, which is sufficient to build a machine-learning model with good accuracy. It combines weather-related data with pollution data. Weather-related data were used as a predictor variable in the proposed study. The study thus correlated the effect of weather data on air quality as well.

In preprocessing, the data were cleaned by noise removal, and the missing values were filled in. Data preprocessing had been done with the following steps:

1. Null values were removed.
2. The wind direction variable had been changed from categorical to dummy (1–0) variables. This is a special preprocessing task that must be carried out on a numerical dataset involving categorical data.
3. Normalization was found to provide better accuracy. Therefore, normalization was used.

While 70% of the air quality dataset was used as the training set, the remaining 30% was used as the test set. The training and testing data remained constant for all iterations as per the parameter settings.

## Experimental result analysis

**Figure 3:**
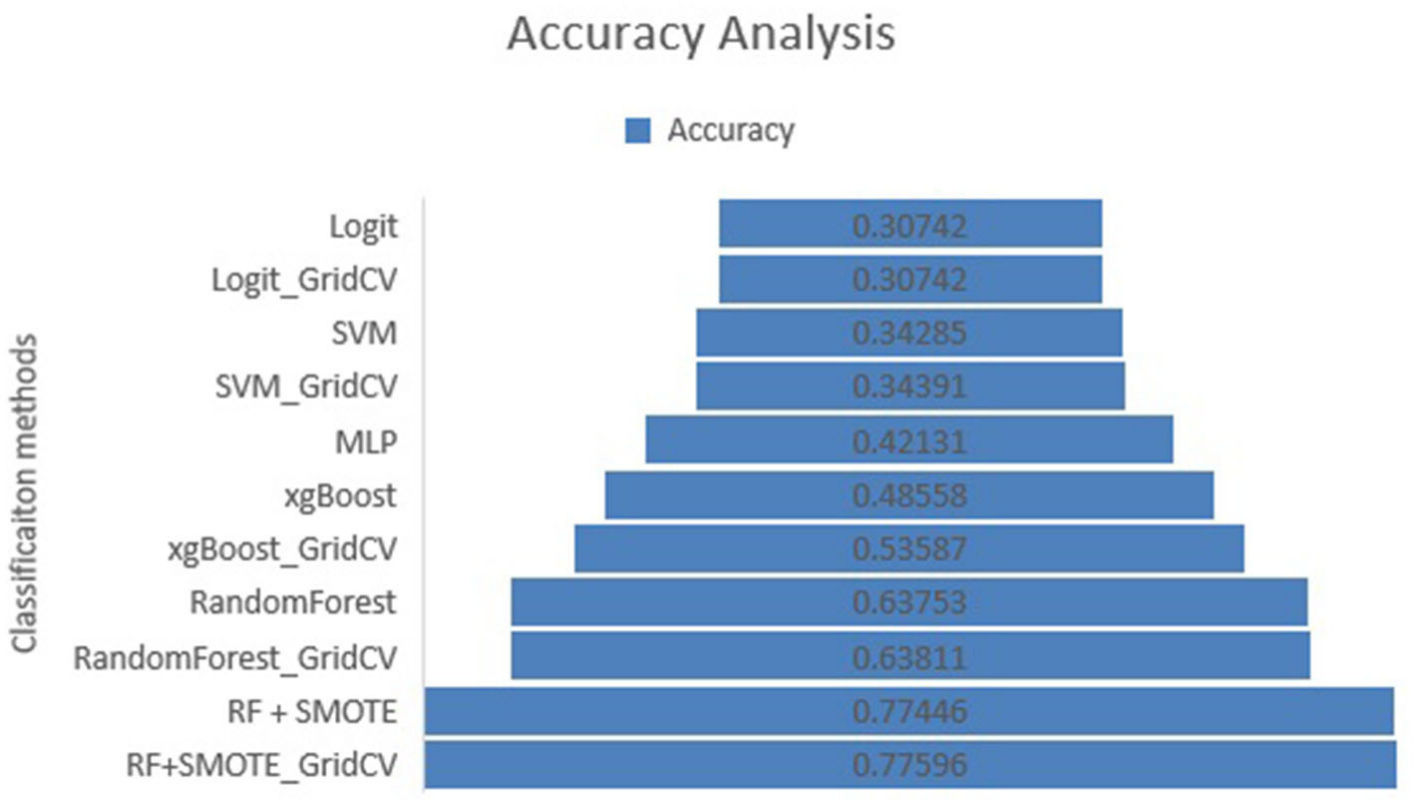
Accuracy analysis for various algorithms used.

In this study, eight machine learning techniques along with MLP were used for building a multiclass classification model of air quality data. The classification accuracy of all the algorithms on the dataset is summarized in Figure 3 for the pollutant PM2.5 for all the algorithms used. This was compared with the proposed multiclass random forest classifier with and without the grid search that uses the SMOTE approach for adding more data to minority classes.

In multiclass classification, imbalanced datasets will have classes that have fewer samples. When classification is performed on an imbalanced dataset using a standard classifier, the model favors the majority class because the majority class has a larger volume of data. The data in the minority class can be oversampled with simple duplication of the samples from the minority class in the training dataset to balance the class distribution. However, this duplication does not provide any new data, as a few of the old data items were duplicated. An added feature to the approach is making new minority-class instances.

The synthetic minority oversampling technique (SMOTE) described in a previous study (Chawla et al., 2002) is the best technique for making new samples. An arbitrary sample belonging to the minority class was chosen first. Then, the *k* nearest neighbors for that sample were found (the typical value of *k* is 5). A synthetic sample was prepared at a randomly chosen point in the feature space between two samples and their arbitrarily chosen neighbor.

Originally, the percentage of rows in the dataset used in this study for each class ranged from 4.44 in the minority class very poor to 33.81 in the majority class good. The percentage of rows in classes becomes even in all six classes after applying the SMOTE.

Figure 3 gives the accuracy analysis for various algorithms for PM2.5 after carrying out our proposed study on the given dataset.

From Figure 3, we can infer that, for the pollutant PM2.5, the accuracy of the proposed RF-SMOTE classifier using the grid search is 77.59%, which is the highest of all the methods used in the analysis.


(1) $$\begin{array}{l} Accuracy=\frac{TP+TN}{P+N}, \end{array}$$

TP refers to true positive, TN refers to true negative, and P and N refer to positive and negative, respectively.

**Figure 4:**
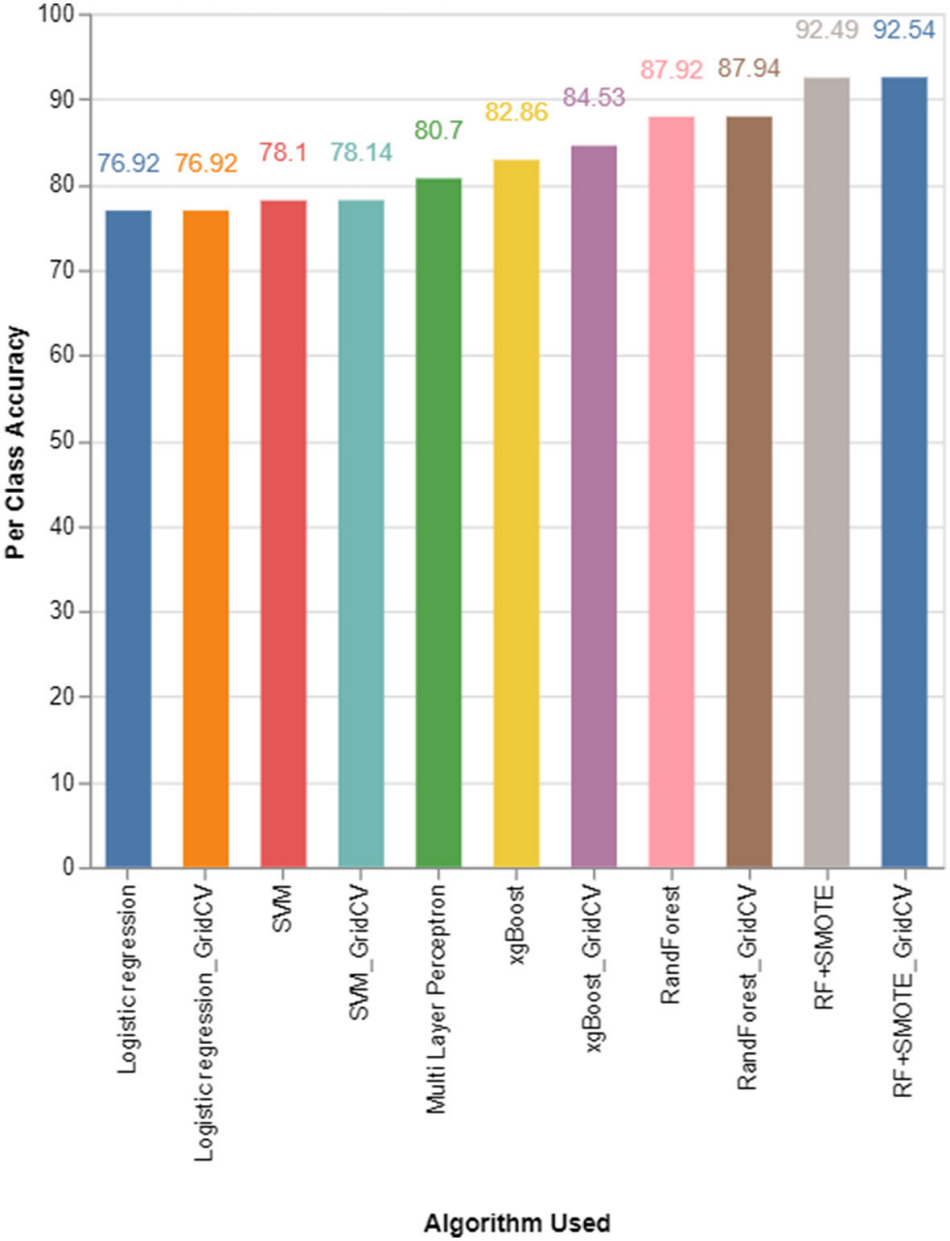
Per-class accuracy comparison for various algorithms used.

Figure 4 and Table 3 demonstrate the comparison of various algorithms for the classification of PM2.5 in terms of the metric per-class accuracy.

**Table 3:** Comparison of per-class accuracy of each algorithm.

**Algorithm**	**Per class accuracy in %**
Logistic regression	76.92
Logistic regression with grid search	76.92
SVM	78.1
SVM with grid search	78.14
MLP	80.7
xgBoost	82.86
xgBoost with grid search	84.53
Random forest	87.92
Random forest with grid search	87.94
RF-SMOTE	92.49
RF-SMOTE with grid search	92.54

The accuracy of one class when compared to all other data points in the dataset is known as the per-class accuracy in multiclass classification. This must be computed for all classes, and their average provides the per-class accuracy. The binary accuracy for each class label must be found separately, and the average value of these binary accuracy values provides per-class accuracy.


(2) $$\begin{array}{l} Per-class\;accuracy=\frac{\sum_{i=1}^{n}Binay\;accuracy\;of\;the\;Class_{i}}{n} \end{array}$$

The definition for per-class accuracy is given above, where *n* is the number of classes.

Figure 4 shows that the random forest classifier using grid search with an accuracy of 87.94% is the best score for the pollutant PM2.5. The least accurate one is the logistic regression classifier, with an accuracy of 76.92%. The accuracy of the proposed RF-SMOTE classifier increased to 92.54% when using a grid search that uses the SMOTE on the dataset for balancing the data across all the classes of the dataset, whereas it was 92.49% for the same algorithm without a grid search.

## Conclusion

In this study, several machine learning techniques have been used to classify the pollutant values of PM2.5 into multiple classes. The dataset used the correlations between weather data and air quality data. Different classifiers have been used to classify the PM2.5 values in this study. Owing to the unbalanced nature of the dataset, a SMOTE-based technique was also recommended, and the findings were reviewed. The machine learning techniques deployed are compared in terms of accuracy and per-class accuracy. The random forest with grid search gives the maximum accuracy score in both the cases with and without SMOTE. Though only the values of PM2.5 were used in the multiclass classification, the method can be used for the forecast of other pollutants as well.

The drawback of the proposed system is that the size of the dataset increases because of over-sampling, and the run time increases many folds as a result. Apart from the SMOTE, various other methods are available for majority-class undersampling or minority-class oversampling. In addition, a lot of variants of the SMOTE are available, such as SMOTE_TomekLinks and SMOTE_ENN (Lu et al., 2019). They can also be deployed on the dataset, and accuracy levels may be compared with the proposed system in future studies.

## Data availability statement

The original contributions presented in the study are included in the article/Supplementary material, further inquiries can be directed to the corresponding author.

## Author contributions

SS created this framework and wrote the article under the guidance of CM. All authors contributed to the article and approved the submitted version.
