# Supplementary material for: Ensemble-based classification approach for PM2.5 concentration forecasting using meteorological data
Source: Front Big Data. 2023 Jun 9;6:1175259. doi: 10.3389/fdata.2023.1175259 (PMC10289837; doi:10.3389/fdata.2023.1175259)
Supplement: Supplementary file 1 [file Data_Sheet_1.docx]

Supplementary Material

Ensemble-based classification approach for PM2.5 concentration forecasting using meteorological data

S. Saminathan^1*^, C. Malathy^2^

^1^Department of Computing Technologies, School of Computing, SRM Institute of Science and Technology, Kattankulathur, 603203, Tamil Nadu, India

^2^Department of Networking and Communications, School of Computing, SRM Institute of Science and Technology, Kattankulathur, 603203, Tamil Nadu, India

*** Corresponding Author**[saminats@srmist.edu.in](mailto:saminats@srmist.edu.in)

**Data availability statement**

The data provided in ([UCI Machine Learning Repository, 2017](https://archive.ics.uci.edu/ml/machine-learning-databases/00501/PRSA2017_Data_20130301-20170228.zip) UCI ML repository) was used in this study. It is cited in the References section of the main article.

# Supplementary Figures and Tables

**
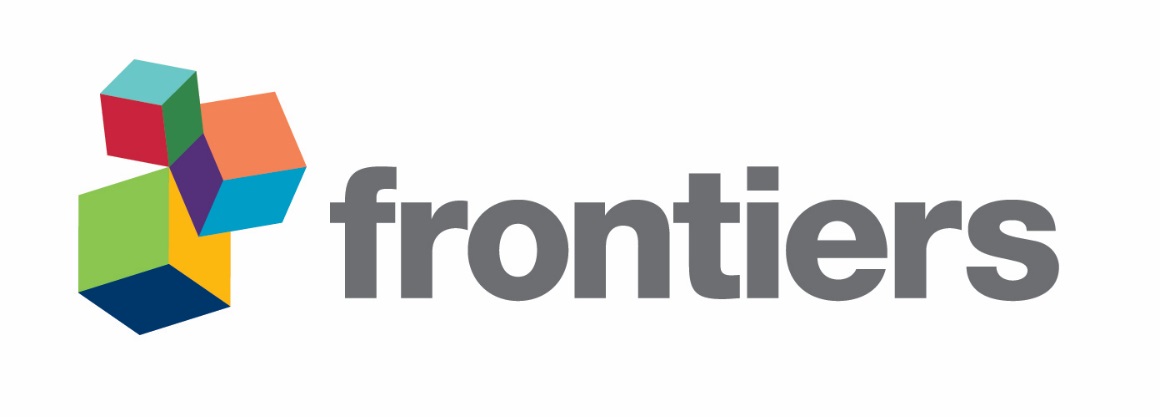
**

Supplementary Figures 1 and 2 respectively give the distribution of rows in each class for PM2.5 in the actual imbalanced data set and distribution of rows in each class for PM2.5 in the data set after applying SMOTE. The values are given in Supplementary Table 1 as well.

**Supplementary Table 1.** Distribution of rows in majority and minority classes

in the data set before and after applying SMOTE

| **Pollutant class** | **% of rows in the**  **original data set** | **% of rows**  **after applying SMOTE** |
| --- | --- | --- |
| Good | 33.81 | 16.67 |
| Moderate | 14.56 | 16.67 |
| Poor | 10.14 | 16.67 |
| Satisfactory | 19.51 | 16.67 |
| Severe | 04.44 | 16.67 |
| Very Poor | 17.55 | 16.67 |


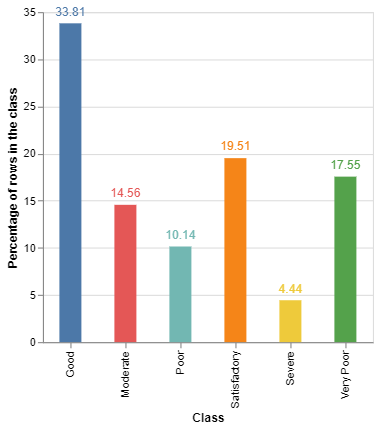


**Supplementary Figure 1** Distribution of rows in each class for

PM2.5 in the actual data set


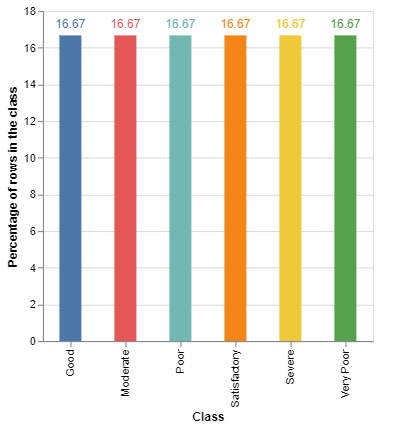


**Supplementary Figure 2** Distribution of rows in each class for

PM2.5 in the data set after applying SMOTE
